# Retinal phototransduction

**Published:** 2014-10

**Authors:** Gurdeep S. Mannu

**Affiliations:** *From the Academic Medicine Department, Norfolk and Norwich University Hospital, Norfolk, United Kingdom*

## Abstract

Vision is perhaps the most important of all our senses, and gives us an immense amount of information regarding the outside world. The initial format in which this information reaches the retina are photons; particles of energy radiation of a given wavelength emitted or reflected from our surroundings. The brain itself however, perceives information in electrical signals via action potentials and changes in electrochemical gradients. The processes involved in the transduction of photons into electrical potentials will be the focus of this article. This review article summarizes the recent advances in understanding these complex pathways and provides an overview of the main molecules involved in the neurobiology of vision.

The eye is a complex structure in terms of cell types. It is due to this complexity that much remains unknown regarding the neurobiology of vision. This article will summarize the current understanding of light transduction within the eye. The eye allows the ‘collection’ and detection of light and may be analogous with a camera. The eye lens focuses light onto an area at the back of the eye called the retina (much like a film or photosensitive chip in a camera). The retina is the most important component of the eye for the detection of all light entering the eye and hence is very specialised in terms of the structure and arrangement of the cells present here. The retina of the eye is composed of 4 different light-sensitive cells divided into rod cells as well as 3 types of cone cell (cone cells being differentiated by the nature of the opsin pigment that they contain). The 4 types of light-sensitive cells, by virtue of their pigments and intracellular machinery, are the focal points of the transduction process, which converts light energy into electrochemical energy. It is due to this function that these cells are termed photoreceptors.^1^ These cells are unique in their design and structure. They are long elongated neuronal cells stacked tightly one next to the other like miniature skyscrapers with their dendritic ends facing in the direction where light is entering the eye. This is followed by a ‘swelling’ in the neurone for the cell’s nucleus. Deeper into the retina is another swelling for the cell’s organelles, especially mitochondria, which cater to the immense energy requirements for the cell. Finally, there is a series of a thousand or so ‘discs’, each side of which containing roughly eighty thousand pigment molecules.^2^ It is these pigment molecules that will be the focus of this article.

The photoreceptor cells are well vascularized with a constant and steady flow of blood, sustaining constant external ion gradients and providing a maintained supply of substrate molecules. The neurone’s dendrites are synapsed upon by bipolar cells, although the exact nature of this synapsing depends on the cell involved. Rod cells particularly show great spatial summation (with one bipolar cell synapsing with many rod cells). Although this summation considerably reduces visual acuity, it does greatly increase visual sensitivity in low light conditions. Cone cells, however, have a one-to-one relationship with their respective bipolar cells, creating high visual acuity, but function poorly in low light conditions. There is also differentiation between the position of both cone cells and rod cells within the surface of the retina itself. Cone cells are more focused; namely, they have a greater concentration in an area known as the fovea (or ‘area centralis’), which is where light is typically focused by the cornea, lens, and refractive fluid within the eye. The fovea requires a greater number of high acuity cells to differentiate and perceive the much larger number of photons being funneled here. Rod cells lie circumferentially and aid with peripheral vision.

The first stage in transducing light into a form the brain can comprehend is the ‘capturing’ of light energy into a chemical format. This is achieved by the presence of various light ‘pigment’ molecules within the outer segments of these receptors. With advances in the theory of wave-particle duality, light is now regarded as being transmitted through space in the form of tiny packets of energy (photons). The energy of each photon varies depending on its frequency (E=hF; where E=energy, F=frequency, h=Planck’s constant). High-energy photons have higher frequencies, and low frequency photons hence have less energy. The higher a photon’s frequency, the lower its wavelength, and different retinal cells are sensitive to particular wavelengths. When photons between 400 to 780nm in wavelength enter the eye, they collide with the pigment molecules inside these retinal cells and cause various conformational changes to occur within them.^3^ These photochemical changes occur as the wavelength of light is converted into its equivalent in chemical energy. These altered molecules are responsible for the intracellular cascade sequence that follows, resulting ultimately in a sequence of electrical signals being sent to the brain.^4^ The brain will receive and process information according to the frequency and pattern of these signals.^5^ This article will describe how light energy is ‘captured’ chemically, converted into electrical energy, and which intracellular machinery, and chemicals are used in this process.

## Literature review

A 2-step process utilizing a Medline/PubMed systematic search was conducted. The initial search was undertaken using elementary phrases including “neurobiology of vision,” “light transduction,” “intracellular mechanism,” and “retinal pigment.” Only the most recent literature in the field was required so the time-window for the literature review was restricted to the past 30 years (1983-2013). The resultant abstracts were analyzed and appropriate papers were selected. The secondary search was performed by (1) using the reference lists of the chosen articles, and (2) by using PubMed weblinks for related articles. The studies were selected if they were in English language and included the appropriate topics. The search produced over 3,300 published papers on the topic of the neurobiology of vision. The abstracts of these were screened and all papers relating to the intracellular signal transduction or mechanisms of action were selected.

## Current status of knowledge

**Figure 1:**
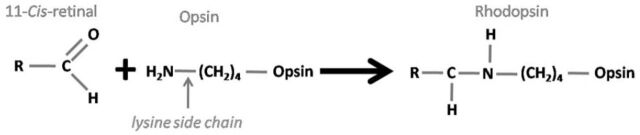
The chemical structure and formation of rhodopsin. C - carbon, H - hydrogen, O - oxygen, N - nitrogen, R - rest of the molecule.

**Figure 2:**
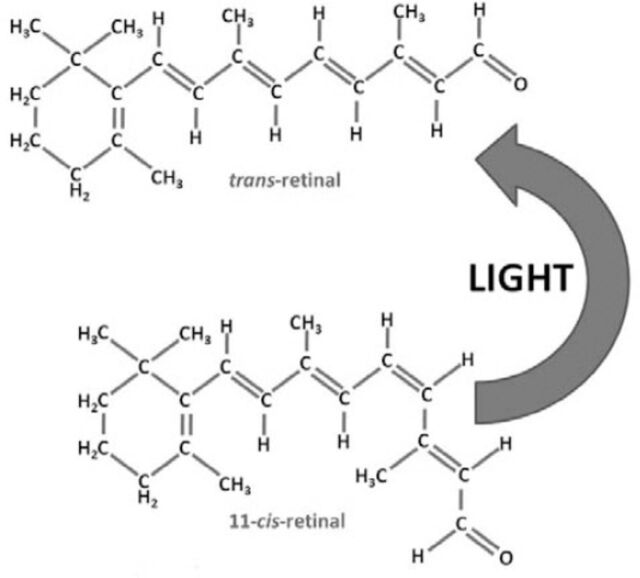
Light causes a conformational change in the retinal molecule from the 11 cis-retinal to the trans-retinal. C - carbon, H - hydrogen, O - oxygen.

Each of the 4 types of photoreceptors has unique pigment molecules which, when absorbing light of a certain frequency, will initiate a distinctive conformational change in structure. For many years the question that interested neuroscientists and researchers in this field was how these different photopigments actually absorbed different frequencies of light. Advances in molecular techniques have revealed that the answer to this lies in the chemical composition of each pigment. All of these individual pigments contain an aldehyde of vitamin A called retinaldehyde (abbreviated to retinal) in addition to one other component called opsin.^6^ It is this opsin that differentiates the different types of photoreceptor.^4^ Rod cells contain rhodopsin (**Figure 1**), which absorbs light with a wavelength of 500 nm (namely, in the blue-green region of light).^7^ However, cone cells contain 3 different pigments, called iodopsins; namely, erythrolabe, chlorolabe, and cyanolabe. Each of these is sensitive to different frequencies of light; erythrolabe is liable for absorption of photons at a wavelength of 565nm (red light), chlorolabe can absorb light of 535nm wavelength (green light), and finally cyanolabe absorbs light of a maximum wavelength of 440nm (in the blue color end of the spectrum).^8^ Despite the variation in the pigments involved, the reaction that follows light interaction with the pigment is consistent. Opsin is bound to retinal by a covalent double bond between a carbon and nitrogen atom to form rhodopsin.^9^ When light contacts the retinal molecule it causes a conformation change to occur at the eleventh carbon atom along the chain (**Figure 2**). This causes the molecule to revert from its 11-cis form, to an 11-trans state.^10^ This change coincides with a change in the molecule’s chemical properties since the 11-trans form does not bind to opsin.

Pathologies can develop at any stage during the light transduction pathway, and thus the pigment molecules are not immune to this. The most common abnormality at this stage of the transduction process is that of ‘color blindness’. Color blindness is a well-established term used to describe the lack of perception of a certain wavelength (or ‘color’) of light. This can result from a defect in the higher stages of the perceptual process, for example, in the lateral geniculate nucleus parvocellular pathway. However, color blindness is most commonly associated with a lack or disruption of one of the 3 opsin pigment molecules found in the cone cells at the very start of the transduction pathway.^11^ Since each of these molecules absorb a different wavelength of light for human trichromatic vision, a lack in any of them would mean perception of that particular range of the light spectrum would be lost.^11^ A problem with each of the pigments results in different pathologies. A defect in the erythrolabe pigment results in ‘protanomaly’ or red-green color blindness. Chlorolabe defects result in ‘deuteranomaly’ (red-green color blindness), and cyanolabe defects result in yellow blue color blindness, or ‘tritanomaly’. Genetic changes in pigment and arteriolar attenuation are seen in retinitis pigmentosa. Rod cells are affected early in the condition and due to the rod cells higher sensitivity to light, the loss of nighttime vision is an early symptom of the condition alongside gradually worsening loss of peripheral vision. Many distinct defects in DNA have been isolated and related to retinitis pigmentosa. Most of these are transmitted by autosomal recessive inheritance; however, a proportion are autosomal dominant (30-40%) and X-linked (5-15%).^12^

## The light-dependent cascade

**Figure 3:**
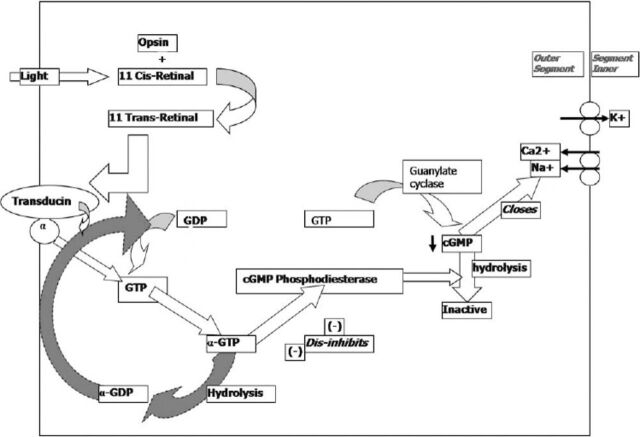
A schematic summary of the light conduction pathway. GDP - guanosine diphosphate, GTP - guanosine triphosphate, GMP - guanosine monophosphate, Na^+^ - sodium ions, Ca^2+^ - calcium ions, K^+^ - potassium ions.

Under dark conditions, photoreceptors are constitutively continuously present in a depolarized state, thus continuously releasing glutamate across the synaptic cleft.^13^ Photons actually result in these photoreceptors becoming hyperpolarized, and as such, signal the changes in electrical potentials in cells. For a change in the electrochemical potential of the photoreceptor cell to occur, ion channels must either be opened or closed. Within the cell, cyclic guanosine monophosphate (cGMP)^14^ is a molecule responsible for closing of sodium and calcium channels.^10^,^15^ The more cGMP there is within the cell, the more channels are closed and the lower the movement of ions and thus reduced electrical activity within the cell. The cGMP is inactivated by the enzyme cGMP phosphodiesterase in a hydrolysis reaction.^16^ Hence, the lower the concentration within the cell of cGMP phosphodiesterase enzyme; the more cGMP will be present and thus, the more ion channels will be closed. However, the more there is of this enzyme, the fewer channels will subsequently close (and there will be more open channels). After contact of light with the retinal molecule, the resulting 11-trans form of retinal can no longer bind to opsin, and thus dissociates from this molecule and moves to the membrane. Here, 11-trans retinal binds to guanosine triphosphate (GTP)-binding protein molecules named transducin, and activates them.^17^ The involvement of transducin is a vital step in the cascade. Transducin consists of 3 subunits; namely, the alpha, beta, and gamma subunits. The alpha subunit contains a molecule of guanosine diphosphate (GDP). The 11-trans retinal causes the alpha subunit to give up its GDP molecule in exchange for a GTP molecule. This exchange causes the entire alpha subunit to dissociate from the rest of the complex,^18^ and to move to interact with the target protein enzyme cGMP phosphodiesterase. Thus, as mentioned earlier this enzyme is responsible for the hydrolysis of cGMP to an inactive form and hence an increase in the influx of sodium and calcium ions (**Figure 3**). Since the photoreceptor remains idle in a constitutively depolarized state, all signals must be signaled by a net hyperpolarization (the exact reverse of what would be the case in many other parts of the human body). At resting conditions (when there is no light), there is an ‘equilibrium’ of ion charge movement. In other words, the net movement of calcium and sodium ions from the interstitial fluid into the outer segment of the photoreceptor is equalized by an outward movement of potassium ions in the inner segment.^19^ As a result, the cell has a net current of around -34pA due to the net flow of calcium and sodium ions through the cGMP-gated transmembrane transporter channel into the outer segment.^20^ When the photoreceptors are exposed to photons; namely, the eyes are present in light conditions; a change in the electrical activity of the cell occurs. Through the guanylate cyclase transduction pathway of intermediates, the calcium/potassium transporter in the outer part of the photoreceptors closes due to the increase in cGMP, and thus; as a result, the influx of positively charged calcium and potassium ions ceases. However, despite this event, the potassium transmembrane transporter is not cGMP gated, and thus remains open. The consequence of this is a continuing outward movement of potassium ions from the photoreceptor into the interstitial environment, and as such, a net decrease in intracellular charge. This net outward flow of singly positive potassium ions reduces the cell potential and hence hyperpolarizes the cell by approximately 1mV, known as the photovoltage.^20^ It is via a lack of depolarization (by hyperpolarization) that information is coded in electrical potentials describing the contact of photons of different frequencies with retinal pigment molecules.

## Deactivation of the cascade

It is necessary for this transduction system to reset itself in order for further signals to be sent. Our environment is ever changing and adapting, and in order for us to successfully survive within these surroundings, we must anticipate and adapt as well. The speed by which we react to changing stimuli from our environment is of vital importance, and this relies on the speed by which we may perceive changes occurring. This perception is dependent on the ‘resetting’ of the transduction cascade to allow it to be reactivated and ‘triggered’ once again. This allows any changes occurring between the 2 inputs in time to be compared and extrapolated by the brain in order to explain what exactly is occurring in the outside world.

An accurate, yet simple analogy of this is a film tape. The tape consists of a series of static, non-moving pictures, which once perceived one after the other at sufficient speed may delude the brain into believing that animation is occurring; this is in fact, exactly what happens continually in human perception of the outside world. The rate at which a static image may be perceived as moving depends on the ‘reset’ time for the light transduction system to reset itself. This is referred to as the critical fusion frequency, and in humans, in bright lighting conditions this may be around 60Hz. It should be noted that in dark conditions, there are much fewer action potentials initiated, and thus the critical fusion frequency is reduced by around 6 times! The resetting of this system involves the deactivation of the 11-trans retinal and its transformation back into its cis- isomer form so as to prevent further production of transducin. This deactivation of 11-trans retinal is carried out by phosphorylating it using an enzyme called rhodopsin kinase. This phosphorylation increases the affinity of arrestin to the rhodopsin molecule,^21^ which inhibits the ability of this molecule to produce further transducin; hence, aiding rhodopsin recuperation. The high intracellular concentrations of Ca^2+^ ions (resulting from the sodium-calcium exchange pump activated by the cGMP that trans-retinal produces), inhibit guanylate cyclase (the enzyme responsible for the synthesis of cGMP from GTP).^22^ As a result, this negative feedback loop limits the transduction cascade. However, calcium ions also inhibit the phosphorylation of rhodopsin by rhodopsin kinase, and so preventing recovery and maintaining the active form of the molecule in an apparent positive feedback loop.^22^

This calcium dependent effect on the activity of rhodopsin kinase occurs due to the involvement of another important regulator protein; so appropriately named, ‘recoverin’. Recoverin binds to the amphipathic peptide at the N-terminus of rhodopsin kinase,^23^ and as long as it is bound, this kinase cannot continue to phosphorylate rhodopsin. This phosphorylation is responsible for halting the transduction cascade by preventing the maintained presence and action of the active form of rhodopsin that will in turn inevitably activate further transducin molecules. Thus, the inhibition of rhodopsin kinase prevents this rhodopsin inactivation, and thus halts the cascade and allows time for rhodopsin to ‘recover’ back into its ‘cis’ optical isomer ‘stand-by’ form.

In conclusion, this article has looked at the major factors and molecules involved in light transduction as well as their interrelationships with each other and the pathways through which they bring about their actions. However, this is a rapidly evolving field with powerful techniques being developed to explore the genetic coding for the molecules involved in the transduction process in recent years. This will no doubt provide us with deeper knowledge of the roles of the molecules described in this article. The fields of signal conduction and signaling in vision are likely to continue to be the source of interest and focused research with an ever-evolving picture of the underlying mechanisms.

## References

[1] Regus-Leidig H, Brandstatter JH (2012). Structure and function of a complex sensory synapse. Acta Physiol (Oxf).

[2] Kennedy B, Malicki J (2009). What drives cell morphogenesis: a look inside the vertebrate photoreceptor. Dev Dyn.

[3] Palczewski K (2012). Chemistry and biology of vision. J Biol Chem.

[4] Terakita A (2005). The opsins. Genome Biol.

[5] Shi G, Yau KW, Chen J, Kefalov VJ (2007). Signaling properties of a short-wave cone visual pigment and its role in phototransduction. J Neurosci.

[6] Nickle B, Robinson PR (2007). The opsins of the vertebrate retina: insights from structural, biochemical, and evolutionary studies. Cell Mol Life Sci.

[7] Tam BM, Moritz OL (2009). The role of rhodopsin glycosylation in protein folding, trafficking, and light-sensitive retinal degeneration. J Neurosci.

[8] Alpern M, Bastian B, Moeller J (1982). In search of the elusive long-wave fundamental. Vision Res.

[9] Jastrzebska B, Debinski A, Filipek S, Palczewski K (2011). Role of membrane integrity on G protein-coupled receptors: Rhodopsin stability and function. Prog Lipid Res.

[10] Wolf G (2004). The visual cycle of the cone photoreceptors of the retina. Nutr Rev.

[11] Neitz J, Neitz M (2011). The genetics of normal and defective color vision. Vision Res.

[12] Hartong DT, Berson EL, Dryja TP (2006). Retinitis pigmentosa. Lancet.

[13] Arshavsky VY (2002). Rhodopsin phosphorylation: from terminating single photon responses to photoreceptor dark adaptation. Trends Neurosci.

[14] Moriondo A, Rispoli G (2003). A step-by-step model of phototransduction cascade shows that Ca2+regulation of guanylate cyclase accounts only for short-term changes of photoresponse. Photochem Photobiol Sci.

[15] Koch KW, Duda T, Sharma RK (2010). Ca(2+)-modulated vision-linked ROS-GC guanylate cyclase transduction machinery. Mol Cell Biochem.

[16] Bereta G, Wang B, Kiser PD, Baehr W, Jang GF, Palczewski K (2010). A functional kinase homology domain is essential for the activity of photoreceptor guanylate cyclase 1. J Biol Chem.

[17] Jastrzebska B, Tsybovsky Y, Palczewski K (2010). Complexes between photoactivated rhodopsin and transducin: progress and questions. Biochem J.

[18] Rang HP, Dale M (2003). Pharmacology.

[19] Bauer PJ (2002). Binding of the retinal rod Na+/Ca2+-K+exchanger to the cGMP-gated channel indicates local Ca(2+)-signaling in vertebrate photoreceptors. Ann N Y Acad Sci.

[20] Kizhatil K, Sandhu NK, Peachey NS, Bennett V (2009). Ankyrin-B is required for coordinated expression of beta-2-spectrin, the Na/K-ATPase and the Na/Ca exchanger in the inner segment of rod photoreceptors. Exp Eye Res.

[21] Kaufman PL, Alm A, Adler FH (2003). Adler's physiology of the eye: clinical application.

[22] Bereta G, Wang B, Kiser PD, Baehr W, Jang GF, Palczewski K (2010). A functional kinase homology domain is essential for the activity of photoreceptor guanylate cyclase 1. J Biol Chem.

[23] Higgins MK, Oprian DD, Schertler GF (2006). Recoverin binds exclusively to an amphipathic peptide at the N terminus of rhodopsin kinase, inhibiting rhodopsin phosphorylation without affecting catalytic activity of the kinase. J Biol Chem.

